# Identification of a large, fast-expanding HIV-1 subtype B transmission cluster among MSM in Valencia, Spain

**DOI:** 10.1371/journal.pone.0171062

**Published:** 2017-02-02

**Authors:** Juan Ángel Patiño-Galindo, Manoli Torres-Puente, María Alma Bracho, Ignacio Alastrué, Amparo Juan, David Navarro, María José Galindo, Concepción Gimeno, Enrique Ortega, Fernando González-Candelas

**Affiliations:** 1 Unidad Mixta Infección y Salud Pública FISABIO-CSISP / Universidad de Valencia and CIBER Epidemiología y Salud Pública, Valencia, Spain; 2 Unidad Prevención del SIDA y otras ITS, Valencia, Spain; 3 Hospital Clínico Universitario-Universidad de Valencia, Valencia, Spain; 4 Hospital General Universitario, Valencia, Spain; Fudan University, CHINA

## Abstract

We describe and characterize an exceptionally large HIV-1 subtype B transmission cluster occurring in the Comunidad Valenciana (CV, Spain). A total of 1806 HIV-1 protease-reverse transcriptase (PR/RT) sequences from different patients were obtained in the CV between 2004 and 2014. After subtyping and generating a phylogenetic tree with additional HIV-1 subtype B sequences, a very large transmission cluster which included almost exclusively sequences from the CV was detected (n = 143 patients). This cluster was then validated and characterized with further maximum-likelihood phylogenetic analyses and Bayesian coalescent reconstructions. With these analyses, the CV cluster was delimited to 113 patients, predominately men who have sex with men (MSM). Although it was significantly located in the city of Valencia (n = 105), phylogenetic analyses suggested this cluster derives from a larger HIV lineage affecting other Spanish localities (n = 194). Coalescent analyses estimated its expansion in Valencia to have started between 1998 and 2004. From 2004 to 2009, members of this cluster represented only 1.46% of the HIV-1 subtype B samples studied in Valencia (n = 5/143), whereas from 2010 onwards its prevalence raised to 12.64% (n = 100/791). In conclusion, we have detected a very large transmission cluster in the CV where it has experienced a very fast growth in the recent years in the city of Valencia, thus contributing significantly to the HIV epidemic in this locality. Its transmission efficiency evidences shortcomings in HIV control measures in Spain and particularly in Valencia.

## Introduction

Contrarily to intravenous drug users (IDUs) and heterosexual people (HT), the number of new HIV diagnosis among MSM in the European Union and European Economic Area (EU/EEA) has increased in the last years [1]. This trend is evident in the particular case of Spain, were IDU was consider the main transmission risk during the late 90s. However, in 2013, 51.2% of the 3278 new HIV diagnoses reported in this country occurred among MSMs [2,3]. One of the factors contributing to this resurgence of HIV infections is the continued increase in unprotected anal sex among MSM that occurs since the highly active antiretroviral therapy (HAART) was introduced in 1996 [4,5]. Molecular epidemiology analyses have revealed the vulnerability of MSM to HIV infection in different ways, such as the frequent detection of transmission clusters affecting this risk group [6–10], and the estimation of shorter times between infections compared to those of HTs and IDUs [11]. In Spain, MSMs have been associated with significantly higher levels of local clustering than other risk groups [11,12]. Also, Delgado et al. [13] recently detected a large HIV-1 subtype F cluster affecting tens of MSM from different Spanish regions, indicating a fast and uncontrolled transmission among recently infected MSM who were unaware of their HIV status.

With approximately 5 million inhabitants, the Comunidad Valenciana (CV) is the fourth most populated region in Spain. Genotypic tests of resistance to antiviral drugs, by sequencing portions of the protease and retrotranscriptase (PR/RT) regions, are performed routinely in the CV. These tests produce large data sets of HIV-1 sequences that can be subjected to evolutionary analyses to better understand the local epidemics. The only molecular epidemiology analyses of HIV in the CV published so far were aimed at reporting the emergence of different CRFs among local MSM [14,15].

By means of phylogenetic analysis, we have identified an exceptionally large HIV-1 transmission cluster mainly localized in the city of Valencia (third largest city in Spain and the capital of the CV, with a metropolitan area of >1,500,000 inhabitants) and which is characterized by a rapid and recent expansion among MSM.

## Materials and methods

### Dataset

**Fig 1:**
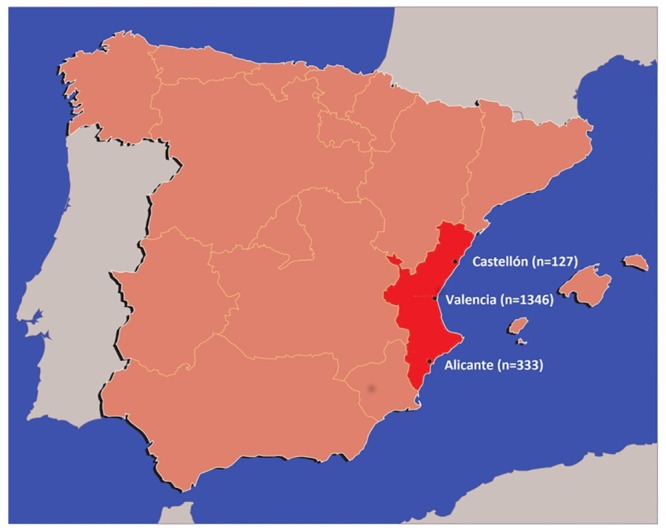
Location of the Comunidad Valenciana in Spain. The Comunidad Valenciana is highlighted in red. The locations of Valencia, Castellón and Alicante, and the number of HIV-1 sequences collected in each of the three localities (from a total of 1806 sequences), are specified.

In order to assess the presence of resistance-associated mutations, 1806 PR/RT sequences were obtained from different newly HIV diagnosed people at seven different hospitals and two HIV counseling and testing centers (CIPS) from the three provinces in the CV between 2004 and 2014: six hospitals (Hospital Clínico de Valencia, Hospital General de Valencia, Hospital Universitario Doctor Peset, Hospital de Manises, Hospital La Ribera, Hospital Francesc de Borja) and one CIPS in Valencia, one CIPS in Alicante and one hospital (Hospital General de Castellón) in Castellón) (Fig 1). The sequences comprised the complete PR and the first 1005 nucleotides (335 amino acids) of the RT (1302 nt in total), and were obtained through viral RNA extraction followed by RT-PCR and direct sequencing using amplification and procedures described previously [16]. All the 1806 newly obtained sequences are available in S1 File. Sequences were subtyped using the REGAv3 [17] and COMET HIV-1 Subtyping tools (http://comet.retrovirology.lu/), and by examination of an initial phylogenetic tree obtained with FastTree 2.1 [18] under the GTR + Γ (4 CAT) model, which included 169 reference sequences downloaded from the Los Alamos HIV Database (LANL; http://www.hiv.lanl.gov), and represented the diversity of HIV-1 group M. Only those sequences classified as subtype B were considered in subsequent analyses. Nucleotide alignments were obtained with MAFFT version 7 [19]. This analysis was part of the surveillance program of communicable diseases by the General Directorate of Public Health of the Comunidad Valenciana and, as such, falls outside the mandate of the corresponding Ethics Committee for Biomedical Research. All personal information was anonymized and no data allowing individual identification was retained.

### Delimitation of the transmission cluster

An initial HIV-1 B phylogenetic tree was reconstructed with FastTree2.1, using the GTR + Γ (4 CAT) model. In addition to the 1514 HIV-1 subtype B sequences from the CV, 133 non-B reference sequences and 1787 HIV-1 subtype B sequences were downloaded from LANL and were included in this analysis. In this tree, a large and highly supported cluster of 143 sequences from the CV was found and further analyzed, given its potential interest within the local epidemic occurring in the CV. A BLAST search for the 100 sequences deposited in GenBank with the highest similarity to each of the sequences in the cluster was performed in October, 2015. This resulted in a non-redundant dataset of 587 BLAST-derived sequences which, along with those from the proposed transmission cluster, 6 subtype B references and 40 additional Spanish sequences (kindly provided by Dr. JC Galán and Dr. M Thomson), were used to reconstruct a maximum-likelihood (ML) tree with PhyML [20]. The resulting tree was used to confirm that these sequences conformed a true transmission cluster (defined as a group of epidemiologically related sequences which share a common, recent ancestor; [21,22], excluding those sequences that were more closely related to those from the BLAST search or additional controls but falling outside the CV clade. Consequently, the criterion used to confirm and delimitate the potential transmission cluster was finding a clade in which more than 90% of its sequences were from the CV and grouped with aLRT support ≥ 0.99.

### Dated phylogeny

The molecular clock signal of the transmission cluster was assessed by performing a linear regression analysis between the parameters “root-to-tip divergence” and “sampling date” with TempEST [23]). We used as input the subtree that included the 143 sequences potentially belonging to the CV transmission cluster, as extracted from the HIV-1 subtype B tree obtained with FastTree. All the 143 sequences from the CV that grouped initially were included in the analysis. The multiple alignment, including time of sampling information, is available in the S2 File.

The most recent common ancestor (tMRCA) of the transmission cluster was dated by means of Bayesian coalescent analysis as implemented in BEAST 1.8.1 [24]. All the 143 sequences from the CV were included in the analysis. For the coalescence analysis, a GTR_112_ + CP_112_ + Γ_112_ (4 CAT) evolutionary model was used and combined with an uncorrelated lognormal relaxed molecular clock model and three different demographic models (Bayesian Skyline Plot, and exponential or logistic demographic change). The best demographic model was chosen using Akaike’s Information Criterion (AICM, [25]). For each demographic model, two independent runs of Bayesian MCMC, with chain lengths of at least 30 million states were performed, and sampled regularly every 3000 generations. These runs were then combined after discarding 10% as burn-in. The evolutionary parameters were estimated from an effective sampling size >200. Trees generated were then summarized using TreeAnnotator (http://beast.bio.ed.ac.uk/).

The internal branch lengths of a transmission cluster can be used as estimates of the time between transmission events [7]. We obtained the distribution of times between transmissions in the cluster from its internal branch lengths, using the summarized BEAST tree.

### Detection of drug resistance mutations

The presence of mutations associated with resistance to PR and RT inhibitors in the transmission cluster was assessed using the Stanford HIV Resistance database (http://sierra2.stanford.edu/sierra/servlet/JSierra; [26]). Only major mutations were taken into account.

### Statistical tests

Univariate analyses (Fisher’s exact tests) were perform in R [27] to check whether the transmission cluster presented a significantly more affected gender (male vs female), transmission risk (MSM vs HT) and/or sampling locality (Valencia vs other localities). Patients from the transmission cluster were compared with the other HIV-1 B patients from the whole dataset.

## Results

**Fig 2:**
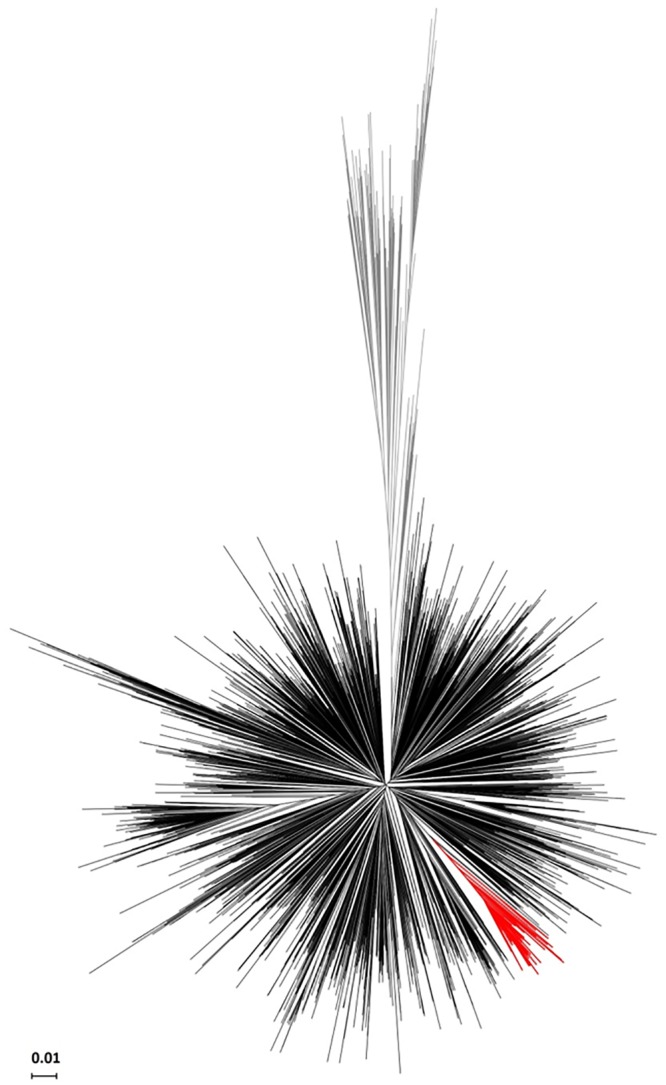
Phylogenetic tree of the HIV-1 subtype B dataset. The potential transmission cluster (n = 143) is highlighted in red, other subtype B sequences are colored in black and the reference sequences from other subtypes/CRFs are colored in grey.

Among the 1806 HIV-1 *pol* sequences obtained from different patients in the CV between 2004 and 2014, 1514 were classified as subtype B (prevalence = 83.83%). A potential transmission cluster was found in the initial HIV-1B tree obtained with FastTree (Fig 2), and it was further validated with a ML tree obtained with 633 additional, non-redundant sequences retrieved in a BLAST search and additional controls as detailed in Material and Methods.

**Fig 3:**
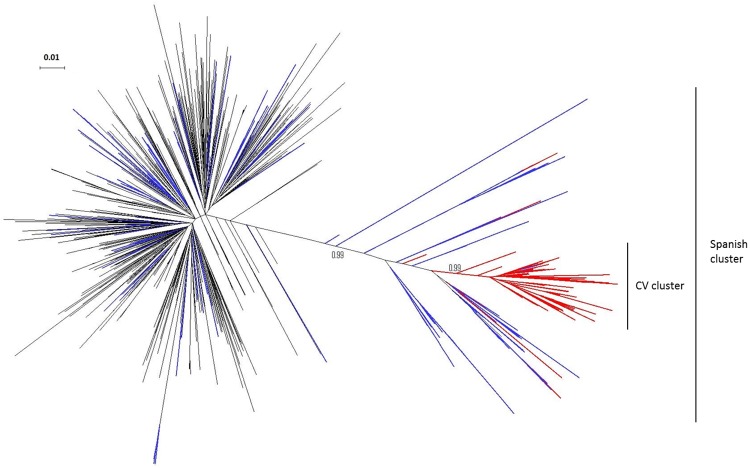
Maximum likelihood tree of the CV-cluster. It includes sequences from the city of Valencia (red), from other Spanish cities (blue) and sequences from other countries retrieved by BLAST analysis (grey). aLRT support values defining the CV-cluster (n = 113) and the larger Spanish clade it derives from (n = 194) are shown.

The ML tree is shown in Fig 3 and it revealed that 111 of the 143 sequences from the CV initially detected as a potential transmission cluster retained monophyly with very high statistical support (approximate Likelihood-Ratio Test, aLRT = 0.99). This reduced cluster, including those 111 sequences, will be referred to as “CV-cluster”. More specifically, the CV-cluster included 105 sequences from patients living in the city of Valencia and its metropolitan area and 6 from patients living in other localities from the CV. Only 2 sequences sampled in other Spanish cities outside the CV were included in this cluster, thus giving a total size for the CV-cluster of 113 patients.

Compared to the full dataset of HIV-1B sequences, the transmission cluster included a disproportionally large number of men [Fisher’s exact test, FET: odds-ratio (OR) = 7.41 (95% CI: 1.94–62.95), P < 0.001], and MSM compared to HTs [FET: OR = 5.63 (1.80–28.40), P < 0.001]. Geographically, the transmission cluster included a very large number of sequences of persons living in the city of Valencia and surroundings compared to other localities [OR = 4.74 (2.29–11.38), P<0.001, Table 1]. The average pairwise genetic distance for sequences in the CV-cluster was 0.0179 substitutions/site (max = 0.0392 s/s; 99 percentile = 0.0308 s/s; standard deviation = 0.0057 s/s).

**Table 1:** Epidemiological characteristics of the patients.

	Cluster (n = 111)	HIV-1 B outside the cluster (n = 1403)
**Sampling location**		
** Valencia**	105	1029
** Alicante or Castellón**	6	374
**Gender**		
** Male**	95	724
** Female**	2	113
** Unknown**	14	566
**Transmission risk** ^a^		
** MSM**	72	515
** HT**	3	121
** IDU**	0	85
** Unknown**	36	682

^a^ MSM:Men who have sex with men; HT: Heterosexual; IDU: Intravenous drug users.

**Fig 4:**
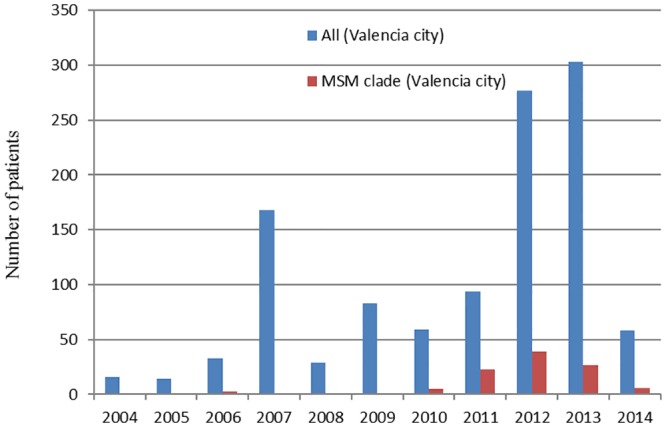
Sampling distribution of HIV-1 subtype B sequences. Sequences from patients inhabiting the city of Valencia or its metropolitan area are represented in blue (n = 1134) and those belonging to the transmission cluster sampled in this city are shown in red (n = 105).

The 111 Valencian sequences from the CV-cluster were sampled between 2006 and 2014, and 105 of them were sampled from 2010 onwards. Considering only those HIV-1 subtype B sequences from the city of Valencia (n = 1134), the transmission cluster accounted for 1.46% of all HIV-1 subtype B sequences sampled in this city between 2004 and 2009 (n = 5/343) but they represented 12.64% of the samples obtained between 2010 and 2014 (n = 100/791) (Fig 4).

**Fig 5:**
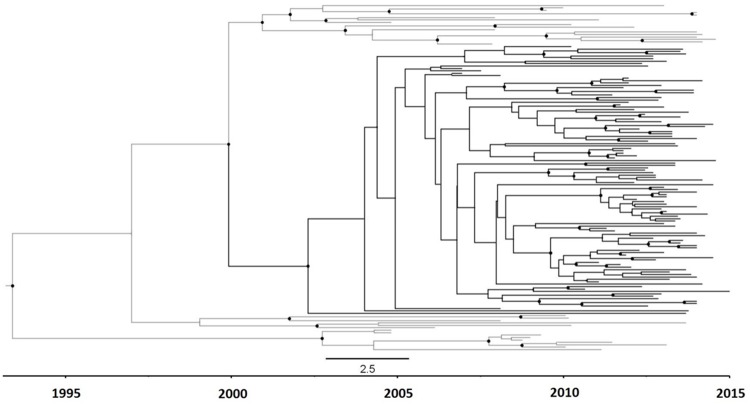
Dated phylogeny of the 143 sequences from the initial CV cluster. Branch lengths represent years. The CV cluster is highlighted in black. Dots on nodes represent posterior probabilities ≥ 0.90.

**Fig 6:**
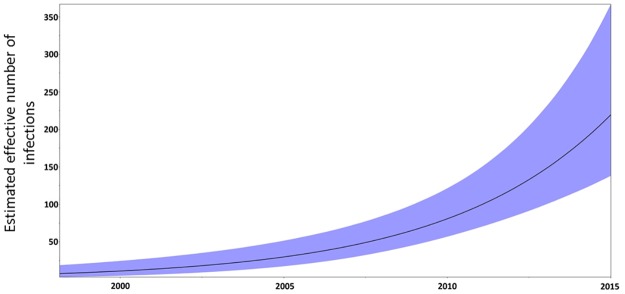
Population dynamics of the 143 sequences from the initial CV cluster. The dynamics was inferred with BEAST, based on the exponential growth model. The black line represents the median estimate of the effective number of infections, and the shaded area represents the 95% HPD.

The clock-like signal present in the analyzed dataset (n = 143) was evaluated by calculating the correlation coefficient (R) between the root-to-tip divergence and sampling date, obtaining an R value equal to 0.61, which was considered high enough as to proceed to estimating the Bayesian dated phylogenies with BEAST [28]. The exponential demographic model, combined with a lognormal relaxed molecular clock yielded the lowest AICM value. The Bayesian coalescent analysis performed under such model estimated the tMRCA of the CV-cluster to have occurred in 2001 (95% HPD = 1998–2004) (Table 2, Fig 5). The median of the internal branch lengths in the Bayesian tree for the CV-cluster was 0.68 years (95% CI = 0.14–3.08). The demographic reconstruction of the CV-cluster shows no indication of deceleration in its growth rate until 2014, the last year of sampling (Fig 6).

**Table 2:** Akaike's Information Criterion (AICM) values and tMRCA of the CV cluster for three demographic models.

Demographic model	AICM ^a^	tMRCA ^b^
**Bayesian Skyline Plot**	17577.24 +/- 0.58	2001.6 (1998.2–2004.6)
**Exponential**	17559.72 +/- 0.38	2001.8 (1998.6–2004.8)
**Logistic**	17585.04 +/- 0.69	2001.9 (1998.8–2004.8)

^a^ (value +/- SE)

^b^ Median and 95% HPD limits

Major resistant mutations were present in very low frequency in the transmission cluster. Major Protease Inhibitors (PIs) resistance mutations were present in only one patient (V82F); major Nucleoside and Non-Nucleoside Reverse-transcriptase Inhibitors (NRTIs, NNRTIs) resistance mutations were present in only another patient (NRTI: M184V + K219E; NNRTI: K103N).

## Discussion

We have detected and characterized an HIV-1 subtype B transmission cluster which, affecting 105 patients solely in the city of Valencia, represents one of the largest local HIV-1 transmission clusters described so far in the HIV pandemic history. The report of clusters of similar or larger size is very rare [8,10,13,29,30], especially those that, like the CV-cluster, affect so many people in a single location in such a short time span.

Although this cluster corresponds to a highly localized HIV-1 outbreak of fast expansion among MSM in the city of Valencia (Spain), phylogenetic analyses revealed that this local cluster is related to sequences sampled in other Spanish cities. Hence, it is likely that, before its introduction and fast expansion in Valencia, this lineage has been circulating in other Spanish localities.

This transmission cluster started its expansion in the city of Valencia around 2001, five years after the introduction of HAART. However, most infections appear to have occurred after 2010, accounting for more than 12% of the HIV-1 subtype B sequences sampled in Valencia since then. This dynamic parallels the increase of HIV diagnosis among MSM in Europe in the last few years [1]. The transmission efficiency of this HIV-1 lineage in the Valencia population, whose growth rate had not reached a steady state by 2014 (last year in our sampling), reveals shortcomings in the HIV control measures in Spain, and particularly in the CV, at least for some specific, vulnerable groups such as MSM.

Previous works have found that recently infected MSMs, who are usually unaware of their HIV status, are a significant source of onward transmissions [31,32] and that, within the MSM collective, the increasing high-risk sexual behavior rates occurring in the last years may hamper the epidemiological benefits of HAART on controlling HIV incidence [4]. In Spain, where there is a high HAART coverage (approximately 74% of the estimated number of persons living with HIV receive treatment; Spain Country factsheet 2015, available at http://aidsinfo.unaids.org), the HIV incidence rate by year of diagnosis for MSMs increased from 2 seroconversions per 100 persons per year (p-y) in 2000 to 2.5/100 p-y in 2009, being the only risk group with a consistently increasing incidence rate along this time-span [33].

Usually, the analysis of transmission clusters is performed after removing major resistance-associated positions from the multiple alignment, in order to prevent spurious clusters resulting from convergent evolution. In this work, these positions were not removed in the identification and characterization of the CV-cluster because most of the sequences included in the analyses derive from treatment-naïve patients. Specifically, none of the 143 patients clustering in the initial group was reported to be or have been under antiretroviral treatment. Furthermore, only two sequences in the CV-cluster presented major resistance mutations. For this reason, we can discard artifactual clustering as a possible explanation for the identification of this cluster.

The criteria used for defining transmission clusters vary among studies, with many of them combining high phylogenetic support with genetic distance thresholds. Although in this work we did not consider large pairwise genetic distances as an exclusion criterion, the distribution of pairwise genetic distances obtained fell within the inclusion criteria of most studies [34]. Furthermore, the maximum distance between any two sequences was lower than 0.045 s/s, a threshold value used previously to consider the exclusion of epidemiologically unlinked sequences [8,9].

The phylogenetic analysis of sequences obtained during routine evaluation of resistance mutations for antiretroviral drugs can provide crucial information about the detailed local, regional and global HIV epidemics [7,9,11]. The identification of transmission clusters is just one of the many possible benefits of the molecular surveillance of infectious diseases, particularly HIV, in which traditional epidemiological analysis based on contact tracing or direct interviews with newly diagnosed individuals are hampered by social and personal attitudes, resulting in lack of useful information for further prevention. This cluster, which was not noticed by local or regional public health officials, was detected and characterized from basic patient information, completely anonymous for the researchers. It also illustrates the need to enhance prevention and information campaigns in a specific risk group.

In conclusion, this work has reported the existence of a large and fast expanding HIV-1 transmission cluster affecting newly diagnosed MSM in the city of Valencia. Given that factors such as high-risk sexual behaviors and unawareness of HIV status may hamper the control of the HIV epidemic in MSM, it is necessary to reinforce the campaigns for HIV prevention, such as condom distribution programs and HIV testing in the Valencian MSM community. The results obtained also stress the importance and interest of implementing surveillance strategies that use viral sequencing information derived from the genotypic analysis of resistance mutations in HIV-infected patients.

## Supporting information

S1 File Fasta alignment of the 1806 HIV-1 sequences obtained from the CV. (RAR)

S2 File Fasta alignment of the 143 HIV subtype B sequences from the CV. These were the sequences initially detected to conform a well-supported cluster and information of their sampling date used in the BEAST analysis is included.
(RAR)
